# Mussel-inspired electroactive, antibacterial and antioxidative composite membranes with incorporation of gold nanoparticles and antibacterial peptides for enhancing skin wound healing

**DOI:** 10.1186/s13036-023-00402-3

**Published:** 2024-01-11

**Authors:** Yongkang Dong, Zheng Wang, Jiapeng Wang, Xuedi Sun, Xiaoyu Yang, Guomin Liu

**Affiliations:** 1 Department of Orthopaedic Surgery, The Second Hospital of Jilin University, Changchun, 130041 China; 2 Department of Vascular Surgery, The Second Hospital of Jilin University, Changchun, 130041 China; 3 Department of Orthopaedic Surgery, Jilin Province FAW General Hospital, Changchun, 130000 China

**Keywords:** Gold nanoparticles, Poly (dopamine), Electrical stimulation, Antimicrobial peptides, Wound healing

## Abstract

Large skin wounds are one of the most important health problems in the world. Skin wound repair and tissue regeneration are complex processes involving many physiological signals, and effective wound healing remains an enormous clinical challenge. Therefore, there is an urgent need for a strategy to rapidly kill bacteria, promote cell proliferation and accelerate wound healing. At present, electrical stimulation (ES) is often used in the clinical treatment of skin wounds and can simulate the endogenous biological current of the body and accelerate the repair process of skin wounds. However, a single ES strategy has difficulty covering the entire wound area, which may lead to unsatisfactory therapeutic effects. To overcome this deficiency, it is essential to develop a collaborative treatment strategy that combines ES with other treatments. In this study, gold nanoparticles and antibacterial peptides (Os) were loaded on the surface of poly(lactic-co-glycolic acid) (PLGA) material through the reducibility and adhesion of polydopamine (PDA) and improved the electrical activity, anti-inflammatory, antibacterial and biocompatibility properties of the polymer material. At the same time, this composite membrane material (Os/Au-PDA@PLGA) combined with ES was used in wound therapy to improve the wound healing rate. The results show that the new wound repair material has good biocompatibility and can effectively promote cell proliferation and migration. Through the combined application of gold nanoparticles and antibacterial peptides Os, the polymer materials have more efficient bactericidal and antioxidant effects. The antibacterial experiment results showed that gold nanoparticles could further enhance the antibacterial activity of antibacterial peptides. Furthermore, the Os/Au-PDA@PLGA composite membrane has good hydrophilicity and electrical activity, which can provide a more favorable cell microenvironment for wound healing. In vivo studies using a full-thickness skin defect model in rats showed that the Os/Au-PDA@PLGA composite membrane had a better therapeutic effect than the pure PLGA material. More importantly, the combination of the Os/Au-PDA@PLGA composite with ES significantly accelerated the rate of vascularization and collagen deposition and promoted wound healing compared with non-ES controls. Therefore, the combination of the Au/Os-PDA@PLGA composite membrane with ES may provide a new strategy for the effective treatment of skin wounds.

**Supplementary Information:**

The online version contains supplementary material available at 10.1186/s13036-023-00402-3.

## Introduction

As the first line of defense in the human body, the skin plays an important role in maintaining electrolyte balance and preventing microbial invasion. Unfortunately, skin is also easily damaged, and skin wounds caused by burns, scalds, lacerations and surgical operations have become one of the most common body injuries [1, 2]. During skin wound healing, factors such as poor circulation, continuous release of proinflammatory cytokines, excess reactive oxygen species (ROS), and bacterial infections often lead to delayed wound healing and may even lead to some syndromes. To solve this problem, researchers have developed a variety of wound healing treatments. In recent years, electrical stimulation (ES) therapy has received much attention for its safety, simplicity and low cost [3]. ES therapy is inspired by the endogenous electric field of organisms, which plays an important role in different stages of wound healing, such as inflammation, proliferation, and reepithelialization [4]. Some studies have found that ES can stimulate keratinocytes to secrete more extracellular matrix by promoting fibroblast proliferation and migration, which can accelerate the wound healing rate [5, 6]. However, traditional ES treatment has difficulty covering the whole wound and requires skin contact to establish a circuit, which is very difficult to do in the treatment of the entire wound area. In addition, the application of ES alone in the treatment of skin injury has limited efficacy. Therefore, developing a cotherapeutic strategy that combines ES with other treatments is critical.

In the treatment of skin wounds, another traditional treatment strategy is to use wound dressings to protect and promote wound healing. Wound dressings can provide a moist wound environment, help absorb exudate and promote tissue regeneration. Researchers have focused on the preparation of wound dressings that promote skin tissue regeneration using different natural and synthetic materials, such as gelatin, chitosan, collagen, silk fibroin and synthetic polymers [7–11]. Among the materials being explored, PLGA and other synthetic polymers have been widely used in recent years. These synthetic polymers have excellent biocompatibility, mechanical properties and adjustable degradation rates and have been widely used to treat various body injuries. However, PLGA and other synthetic materials also have many defects, such as low cell affinity, poor hydrophilicity, and inflammatory reactions caused by degradation products, which seriously limit the further application of PLGA in the field of wound repair [12, 13]. In particular, synthetic polymer materials are generally nonconductive and cannot effectively enhance the tissue induction function of ES when used in combination with ES. Therefore, it is often necessary to modify the surface function of polymer materials to improve their biological activity and electrical activity. For example, many studies have prepared conductive scaffolds for tissue damage repair by modifying polymer materials with conductive materials such as reduced graphene oxide (rGO), carbon nanotubes, and polyaniline [4, 14, 15]. However, the conductivity of such conductive polymers is lower than that of metal-based biomaterials. In contrast, gold has broader applications in material functional modification due to its excellent electrical properties, mechanical robustness and chemical inertness.

Gold nanoparticles have many excellent characteristics, such as small size, large surface area, high reactivity to living cells, good cell permeability, etc., which have attracted increasing interest in wound nursing and treatment [16, 17]. Although gold nanoparticles had weaker antibacterial activity than silver nanoparticles, they had stronger antioxidant and anti-inflammatory activities and were also used as wound dressings. Current studies have demonstrated that gold nanoparticles can effectively reduce wound inflammation and accelerate wound healing due to their antioxidant and anti-inflammatory properties [18]. At the same time, gold nanoparticles can regulate the secretion of proteins (IL-8, IL-12, VEGF and TNF-α) through proangiogenic and anti-inflammatory activities, which are important proteins involved in wound healing [19, 20]. Furthermore, gold nanoparticles show versatility in loading, transporting and unloading various drugs in vivo, and gold nanoparticles have catalytic effects to improve antibacterial properties when combined with other antibacterial substances [16, 21, 22]. At present, there are several commonly used methods to modify tissue repair materials by gold nanoparticles, such as sputtering coating and physical/chemical vapour deposition. However, these methods often require complex instrumentation and are sometimes not compatible with polymers. Recently, some studies have realized in situ fixation of gold nanoparticles through PDA-assisted reduction and fixation [16]. Under alkaline conditions, dopamine can self-polymerize on solid surfaces such as metals, ceramics and polymers to form a polydopamine (PDA) layer [23]. The newly formed PDA layer can reduce the metal ions in solution to gold nanoparticles and fix them on the PDA layer surface. Compared with previous surface modification methods, this method is relatively simple, stable, effective and mild in modification conditions. More importantly, the PDA layer can not only accomplish the in situ synthesis and loading of gold nanoparticles but also effectively improve the hydrophilicity and cell affinity of polymer materials, which further improves the tissue repair ability of the polymer materials.

In the treatment of skin wounds, wound infection is the main cause of delayed wound healing. Thus, another key point of treatment is to prevent wound infection. Traditional antibiotics are effective in treating skin infections; however, long-term use of antibiotics can also lead to some complications, such as imbalances in microbial ecosystems and drug resistance in bacteria [24]. Antimicrobial peptides are components of the innate immune system of all living organisms, which have a wide spectrum of antimicrobial activity and can reduce the incidence of bacterial resistance [25, 26]. Therefore, AMPs are considered promising candidates for the development of novel anti-infective drugs. Os, a 22 amino acid sequence of a defensin from the soft tick Ornithodoros savignyi, was found to kill a variety of bacteria at low concentrations and has excellent biocompatibility [27]. Furthermore, this antibacterial polypeptide also has other therapeutic properties, such as anti-inflammatory and antioxidant activity. Therefore, these antimicrobial peptides can be used as highly effective bioactive factors to modify the surface of polymer dressings to prevent infection and promote wound healing. However, the poor hydrophilicity of polymers and the instability of polypeptides often lead to low loading efficiency. Here, another advantage of dopamine is shown. PDA has many functional groups and strong adhesion ability, which can improve the binding ability of substrate and polypeptide substances. Numerous studies have found that PDA coatings can be used as intermediates for coupling biomolecules to the surface of polymer materials [11, 28]. Thus, the PDA layer can perfectly solve the defect of the low drug loading rate. More importantly, if PDA is used to simultaneously load gold nanoparticles and antibacterial peptides, the performance of antibacterial peptides may be further improved, thus further accelerating wound healing. Therefore, the combination of highly conductive and biocompatible gold-based dressings with antimicrobial peptide delivery is expected to synergistically promote tissue regeneration efficiency.

**Fig. 1:**
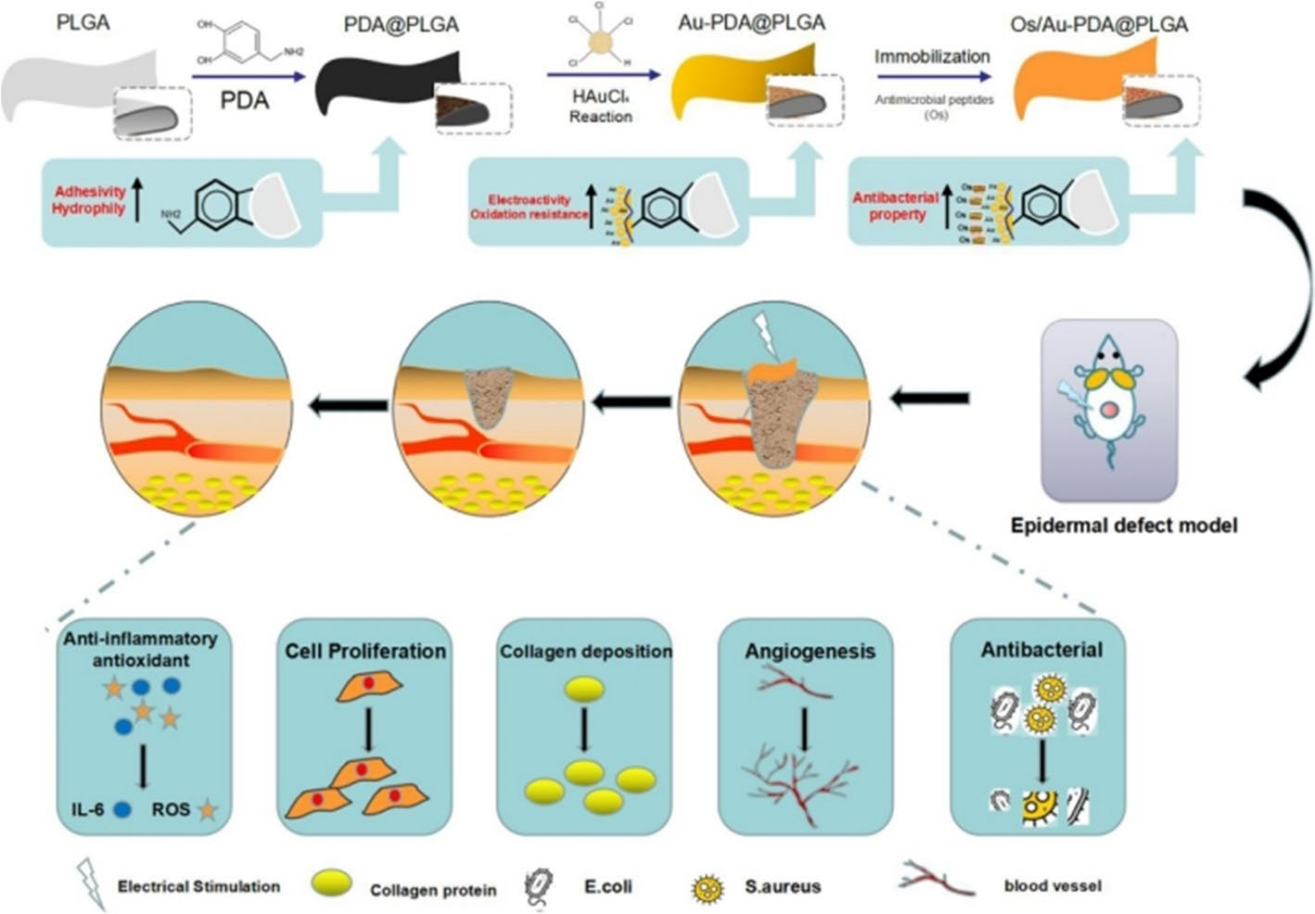
A brief schematic drawing of the preparation and application of the Os/Au-PDA@PLGA composite membrane for skin wound repair

Based on the above considerations, in this study, we first used a PDA layer to modify the PLGA material and loaded gold nanoparticles (Au) and antimicrobial peptides (Os) on the surface of the PLGA material by the reducibility and adhesion of PDA. The above surface modification methods were used to simultaneously improve the biocompatibility, electrical activity, oxidation resistance and antibacterial properties of the PLGA materials to prepare a multifunctional electroactive dressing (Os/Au-PDA@PLGA). Finally, this multifunctional electroactive dressing was used in combination with ES therapy to increase the rate of skin wound healing. A detailed schematic diagram of the dressing material preparation and its application for wound treatment is shown in Fig. 1. The basic purpose of this study is to make possible the sustained synergistic effect of multifunctional electroactive dressing and ES therapy and analyse how their synergistic action may be beneficial in wound healing in terms of antibacterial, antioxidant, and anti-inflammatory activities.

## Materials and methods

### Materials

PLGA (molecular weight = 80,000, LA/GA = 75/25) was obtained from Changchun Institute of Applied Chemistry. HAuCl_4_·3H_2_O and dopamine hydrochloride were purchased from Aladdin Reagents Co., Ltd, China. The polypeptides Os and fluorescein isothiocyanate (FITC)-conjugated Os (FITC-Os) were synthesized by GL Biochem (Shanghai) Co., Ltd. The reagents for cell experiments were purchased from Sigma‒Aldrich (USA).

### Preparation of Au/Os-PDA@PLGA composite membranes

First, 400 mg PLGA was added to 10 ml methylene chloride. After the PLGA was completely dissolved, the PLGA solution was evenly applied to the surface of the glass sheet. After methylene chloride was completely removed, the solidified PLGA membrane was stripped from the surface of the glass sheet. Subsequently, the PLGA membrane was immersed in 2 mg/mL dopamine solution at room temperature for 24 h. After the reaction, the PDA-modified PLGA membrane (PDA@PLGA) was removed and washed with deionized water 3 times. To prepare gold nanoparticles and fix them on the surface of the PDA@PLGA membrane, the PDA@PLGA membrane was immersed in HAuCl_4_·3H_2_O solution (2.0 mM) and reacted overnight at room temperature. The prepared membrane material (Au-PDA@PLGA) was then completely rinsed with deionized water to remove the unreduced gold ions. Afterwards, the Au-PDA@PLGA membranes were immersed in Os (50 μg/mL) solutions for 12 h at 4 °C. Finally, the prepared membrane materials (Os/Au-PDA@PLGA) were completely washed with deionized water to remove the unbinding peptides. All the membrane materials were freeze-dried for 24 h.

### Composite membrane characterization

The surface of each membrane material was treated with gold spraying. The surface microstructure of the membrane materials was observed by scanning electron microscopy (SEM, FEI, Japan). The chemical compositions of the composite membranes were detected by X-ray photoelectron spectroscopy (XPS, Kratos, UK). Composite membrane samples were prepared into sheets of 10 cm and 5 cm width, and a multifunctional mechanical tester (Instron 1121, UK) was used to detect the mechanical properties of the composite membrane samples (Sampling rate: 5 Hz. Maximum tensile strength: 1kN, Drawing rate: 5 mm/min). The hydrophilicity of the membrane sample surface was measured by a water contact angle tester (Kruss DSA 10, GER). The in vitro swelling rate of different membrane materials was determined by measuring the mass change of different samples before and after PBS immersion. The membrane sample (Wd) of the same quality was weighed, and then the sample was soaked in PBS at 37℃ and pH 7.4 for 24 h. After the sample was taken out, the sample was weighed (Ws). The swelling ratio is calculated as follows: swelling (%) = (WS-wd)/Ws. The electrical conductivity of the composite membrane samples was measured by the double probe method (34450A Digital Multimeter, Keysight Technologies, USA). To test the adsorption capacity of PLGA, PDA@PLGA and Au-PDA@PLGA films for Os polypeptides, different samples were immersed in FITC-Os solution for 12 h and then washed with deionized water. Fluorescence images of different membrane samples were obtained using a fluorescence imager (CRI Maestro). Maestro 2.4 software was used to take images and measure average signals.

### Determination of antibacterial activity

*Staphylococcus aureus* (*S. aureus*, ATCC25923) and *Escherichia coli* (E. *coli*, ATCC25922) were used as strains to evaluate the antibacterial properties of the composite membrane. All bacterial strains were provided by the Experimental Center of the Second Hospital of Jilin University. Briefly, composite membranes were first immersed in 75% ethanol solution and sterilized with UV. The sterilized membranes were then rinsed repeatedly with PBS to remove residual ethanol. The bacterial concentration was adjusted to 4 × 10^6^ CFU/mL and cultured in a constant temperature incubator at 37 °C for 2 h. Then, the bacterial solution was cocultured with the sterilized composite membranes. After 10 h, 60 µL of bacterial liquid was absorbed and diluted. Then, 30 µL of diluted bacterial solution was evenly spread onto an LB agar plate and incubated at 37 °C overnight. Finally, the colonies in the LB agar plate were photographed. At present, many studies have proven that PLGA materials have no obvious antibacterial properties, so PLGA samples were selected as the control group. To further evaluate the antibacterial activity of the Os/Au-PDA@PLGA composite membranes in vivo, different membrane materials were implanted into the skin wounds of rats. Then, 50 µl of a mixed bacterial solution of E. *coli* and *S. aureus* (10^6^ CFU bacteria/ml) was added to the rat skin wound. After 3 days, the animals were killed, and the surrounding skin tissue containing membrane material was removed. Finally, the inflammatory reaction of skin tissue was observed by hematoxylin and eosin staining (H&E).

### Antioxidative activity test

To test the antioxidant capacity of the Os/Au-PDA@PLGA composite membranes. We first used a reactive oxygen species (ROS) assay kit (DCFH-DA) to investigate the scavenging ability of different membrane materials on intracellular ROS. Briefly, NIH3T3 cells were inoculated in 24-well plates for 2 h (5 × 10^4^ cells/ml), and then different membrane materials were added to the cell medium. The cells were then cultured for another 24 h. After the medium was removed, the cells were stimulated with 100 μM H_2_O_2_. Finally, the cells were stained with DCFH-DA, and the intracellular ROS were observed using a fluorescence scanning microscope (TE2000-U, Nikon). To further test the antioxidant capacity of different membrane materials. We constructed a wound model on the back skin of rats and implanted different materials into the wound. The rats were killed 2 days after injury, and the skin tissue of the wound was removed and stained with the fluorescent probe DHE. Finally, ROS produced in the skin wounds of rats were observed by fluorescence microscopy.

### Cell proliferation, migration and adhesion assays

NIH3T3 cells (Shanghai Institute of Cell Biology) were used as model cells to evaluate the bioactivity of the ES and composite membranes. Cells were cultured in high-glucose DMEM with 10% foetal bovine serum, 63 mg/L penicillin and 100 mg/L streptomycin at 37 ℃ with 5% CO_2_, and the culture medium was refreshed every 2 days. First, CCK-8 was used to investigate the effects of different ES voltages and stimulation times on the proliferation of cells. Platinum electrodes were then placed on a 24-well plate to create an electric field. A functional signal generator is connected to the electrode to form a signal source. The output signal is monitored by the digital oscilloscope of the signal generator. NIH3T3 cells were inoculated into 24-well cell culture plates at a density of 2 × 10^4^ cells/well. At each specific time point, the medium was replaced with Cell Count Kit-8 and incubated at 37 °C for 2 h. Finally, 100 μl of solution was transferred to a new 96-well plate, and absorbance was measured at 450 nm using a multifunctional microplate scanner (TE2000-U, Nikon). To test the effect of ES combined with different composite membranes on cell proliferation, the composite membrane was immersed in 75% ethanol solution and sterilized by UV. Then, the composite membranes were thoroughly rinsed with PBS and immersed in cell culture medium. Finally, the cells were seeded at a composite membrane with or without electrical stimulation in a 24-well plate at a density of 2 × 10^4^/well. On day 4, CCK-8 assays were used to determine cell proliferation on different composite membranes.

To evaluate cell adhesion, the adherent cells were cultured on various composite membranes, and cells were fixed with 4% paraformaldehyde after a 4-day culture. Subsequently, FITC and DAPI were used to stain the cells. After the cells were washed with PBS, the morphology of the cells was observed by a fluorescence microscope. Cell migration was measured using the wound healing test. The cells were seeded at the composite membrane with or without electrical stimulation in a 24-well plate. When the cell confluence reached 90%, the cells were scratched with a 1000 µl pipette tip. The cells were washed with PBS to remove dead cells. Images of the cells were taken using a fluorescence microscope at different time points. Wound contraction was quantified from the images using the following equation:

$$\mathrm{Wound contraction }(\mathrm{\%})=({{\text{W}}}_{0} - {{\text{W}}}_{t})/{{\text{W}}}_{0} \times 100\mathrm{\%}$$

where W_0_ is the wound area immediately after the wounding procedure and W_t_ is the wound area after time “t” of sample treatment.

### In vivo wound healing assessment

A full-thickness skin defect model was used to characterize the accelerated wound regeneration ability of the composite membranes and ES. Briefly, all animals were anaesthetized with pentobarbital sodium (50 mg kg^−1^) intraperitoneally, and the back hair was removed with a razor and hair removal cream. Then, a circular incision with a diameter of 10 mm was created on the back of the rat, and different composite membranes (PLGA, PDA@PLGA, Au-PDA@PLGA, Os-PDA@PLGA, Os/Au-PDA@PLGA and Os/Au-PDA@PLGA + ES) were implanted into the defects and fixed with gauze. After implantation at 0, 5, 9 and 12 days, the composite membranes were changed, and the wound was observed with a digital camera. The rats in the Os/Au-PDA@PLGA + ES group were treated with 200 mV and 100 Hz electrical stimulation on both sides of the injured skin every day after surgery, lasting for 1 h. The healing rate of the wound was calculated according to the following formula:

$$\mathrm{Wound closure rate }(\mathrm{\%})=({{\text{A}}}_{0}-{{\text{A}}}_{t})/{{\text{A}}}_{0}\times 100\mathrm{\%}$$

A_0_ represents the initial area of the wound, and At represents the area of the wound on days 0, 5, 9, and 12.

### Histological evaluation

On postoperative day 12, skin tissue samples were removed and fixed with paraformaldehyde. The skin tissue was then dehydrated with ethanol, embedded in paraffin blocks and sliced. The tissue sections were stained with H&E, Masson trichrome and Sirius Red, and re-epithelialization and collagen formation were analysed. Meanwhile, the wound sections were immunofluorescently stained with VEGF to evaluate angiogenesis. The wound sections were stained with IL-6 immunohistochemistry to observe the local inflammatory response at the implanted sites. To test the biosafety of different composite membranes, composite membranes were implanted under the skin of rats. One month later, healthy SD rats without surgical treatment were used as the control group. Vital internal organs such as the heart, liver, lung, spleen and kidney were removed and stained with H&E. Finally, the physiological structure of important organs in the experimental animals was observed under a microscope.

### Statistical analysis

All quantitative data were analysed using Origin 8.0 software (Origin Lab Corporation, USA) and expressed as the mean ± standard deviation. Multiple group comparisons were analysed by one-way ANOVA. A value of *p* < 0.05 was considered statistically significant.

## Results and discussion

### Characterization of the Au/Os-PDA@PLGA composite membranes

**Fig. 2:**
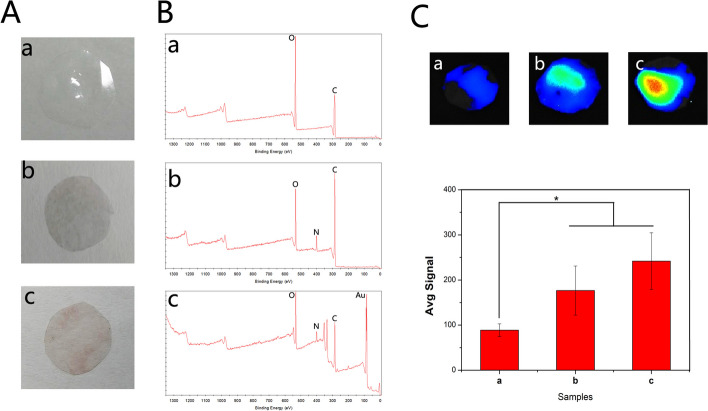
(**A**) Macroscopic images and (**B**) XPS patterns of the PLGA (a), PDA@PLGA (b) and Au-PDA@PLGA (c) membranes. (**C**) The adsorption ability of antibacterial polypeptide Os binding to the PLGA (a), PDA@PLGA (b) and Au-PDA@PLGA (c) membranes, *p* < 0.05, *n* = 3

In this study, inspired by mussel chemistry, we proposed a feasible approach to obtain electroactive, antibacterial and antioxidative skin repair materials. We first constructed a PDA coating on the surface of the PLGA material and then used the adhesion and chemical reducibility of PDA to simultaneously load gold nanoparticles and the antibacterial polypeptide Os on the surface of the material. First, macro-scopic appearance of different composite membrane were shown in Fig. 1A. PLGA is transparent film, and the surface color of the material becomes black after PDA modification. When the gold nanoparticles were loaded on the surface of the material, the surface of the membrane material showed a light red color. Subsequently, we examined the elemental composition of the composite membrane. As shown in Fig. 2B, XPS results show that specific N peaks can be detected in the PDA@PLGA membrane compared with PLGA, indicating that polydopamine was successfully deposited on the surface of the materials. The XPS spectra of Au-PDA@PLGA show a newly formed Au peak at 90 eV, indicating that the gold ions are successfully reduced and deposited on the surface of the material. After the Au-PDA@PLGA composite membrane was prepared, the antibacterial polypeptide Os was loaded into the composite membrane by a physical adsorption method. At present, many studies have enhanced the biological activity of polymer materials by loading growth factors, drugs, antibacterial peptides and other biologically active factors [29, 30]. However, polymer materials lack biomolecular binding sites and hydrophobicity, which often results in a low loading rate of bioactive factors and the explosive release of bioactive factors. One of the main purposes of this study was to enhance the binding ability of the PLGA material to the antibacterial polypeptide Os by PDA and gold nanoparticles to avoid the explosive release of Os. To examine the antibacterial polypeptide-binding ability of different composite membranes, the PLGA, PDA@PLGA and Au-PDA@PLGA membranes were incubated with FITC-O solution. As shown in Fig. 2C, the binding ability of Os to different membranes was detected using an immunofluorescence assay. The average signal intensity represented the amount of binding protein on the membrane. The results showed that the binding ability of Os to the PLGA membrane was lowest due to the weak interaction between Os and PLGA. The fluorescence intensity of the PDA@PLGA and Au-PDA@PLGA membranes was significantly higher than that of the PLGA group, especially the average signal intensity of the Au-PDA@PLGA group, which was more than 2 times higher than that of the PLGA group, showing that Au-PDA@PLGA has better binding ability to Os.

**Fig. 3:**
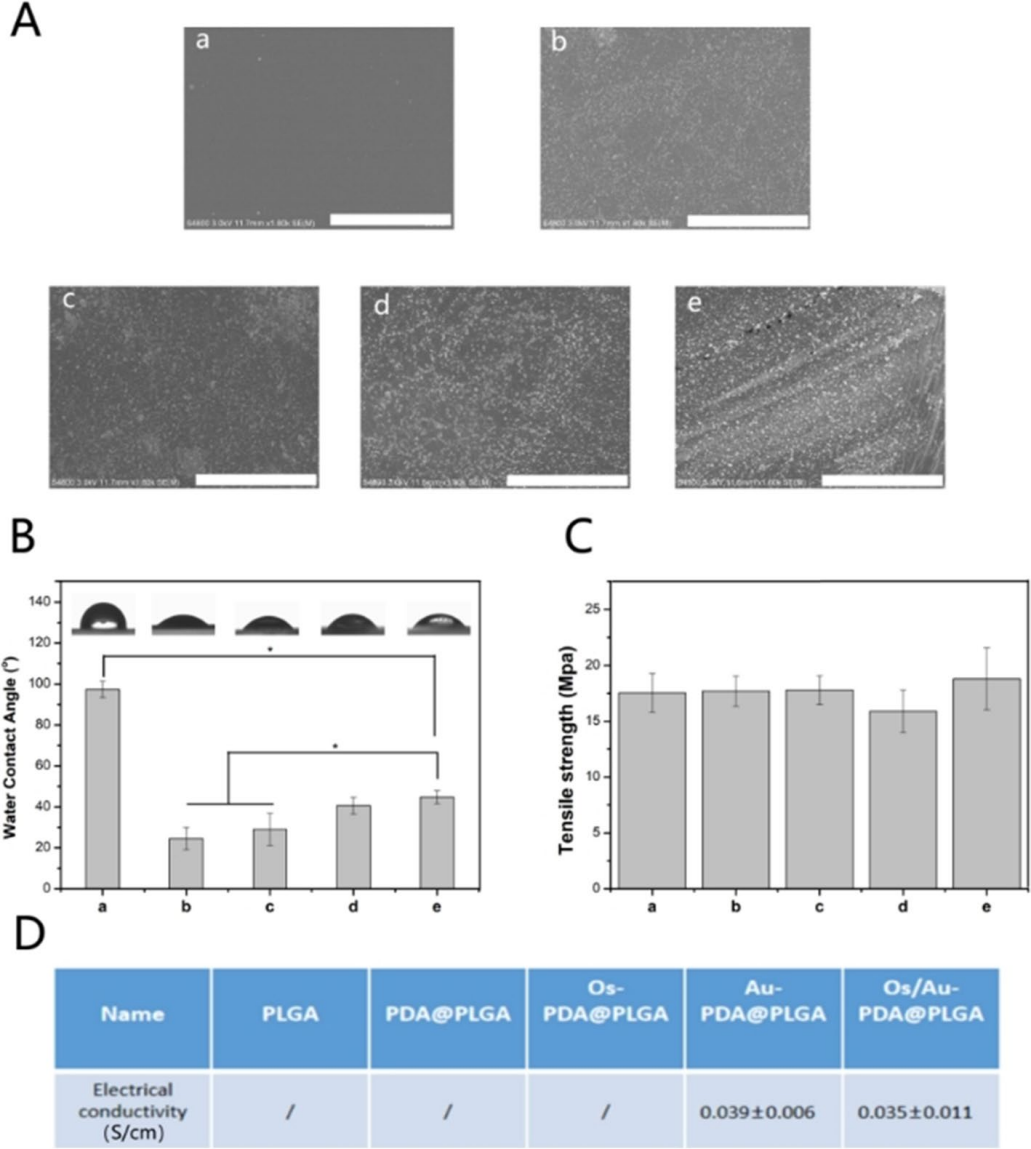
(**A**) SEM images, (**B**) water contact angle, (**C**) mechanical tensile results and (**D**) electrical conductivity results of the PLGA (a), PDA@PLGA (b), Os-PDA@PLGA (c), Au-PDA@PLGA (d) and Os/Au-PDA@PLGA (e) membranes. *p* < 0.05, *n* = 3, Bar lengths are 30 μm

In the process of applying wound repair materials, the surface morphology of materials directly or indirectly affects their biological functions. The surface morphology of different membrane materials obtained by scanning electron microscopy (SEM). Figure 3A shows that the PLGA membrane presents a smooth and flat surface. The surface of the PDA@PLGA membrane was rougher than that of the PLGA membrane. For the Os/Au-PDA@PLGA membranes, the surface is rougher and more uneven, and particles of different sizes can be found attached to the surface. Compared with the Au-PDA@PLGA membrane, the surface morphology changes of the Os/Au-PDA@PLGA membrane are not obvious, which may be related to the small amount of loaded Os. Through SEM observation, PDA and gold nanoparticles can significantly improve the surface roughness of PLGA materials. This rough surface structure can provide more adhesion sites for cells and improve the adhesion behavior of cells to some extent.

The surface hydrophilicity of materials is one of the important parameters that affects the biological activity and repair effect of biomaterials. The hydrophilic surface can effectively promote cell infiltration and adhesion. Meanwhile, if wound dressings are hydrophilic, they will keep the area around the wound moist, which will facilitate healing. In this study, we evaluated the wettability of different membrane materials by measuring droplet contact angles on different material surfaces. As shown in Fig. 3B, the results show that the surface contact angle of the PLGA membrane is the largest, which is 97.4 ± 4.1°. The surface contact angle of the PDA@PLGA membrane (24.6 ± 5.4°) is obviously lower than that of the PLGA membrane, and the hydrophilicity of the material is better. Compared with PDA@PLGA, the Au-PDA@PLGA membrane (40.6 ± 4.17°) has a higher surface contact angle and lower hydrophilicity, but its hydrophilicity is still better than that of PLGA. Compared with the Au-PDA@PLGA membrane, the water contact angle of the Os/Au-PDA@PLGA membrane had no significant change. Some studies have found that when the contact angle of the material is between 0° ~ 40°, the adhesion ability of cells on the material surface is significantly improved [31]. The surface contact angle of the Os/Au-PDA@PLGA membrane was 44.8 ± 3.2°, which may have created a more suitable environment for skin wound healing. Moisture retention is also an important evaluation index of wound repair materials. The moisture retention performance of a material is generally determined by its water absorption. In this study, the water absorption performance of different membrane materials was analyzed by testing the swelling rate. Figure S1 shows the swelling rate of different spun fiber membranes in PBS within 24 h. The swelling rate of pure PLGA membrane was the lowest. After the material was modified by PDA, the swelling rate of the membrane material increased obviously. When the gold nanoparticles and Os are loaded on the surface of the material, the swelling rate of the material did not change much. The results show that the swelling rate of the material has a strong consistency with its surface hydrophilicity. In this study, we also analysed the tensile strength of different membrane materials by mechanical testers. As shown in Fig. 3 C, the tensile strength of the pure PLGA membrane was 17.53 ± 1.75 MPa. After the addition of PDA, gold nanoparticles and Os, the tensile strength of the membrane did not change significantly, so the addition of PDA, gold nanoparticles and Os had little effect on the mechanical properties of the membrane materials. Next, we compared the conductivity of different membrane materials. The conductivity of the membrane was measured by using the double probe method (Fig. 3D). The results showed that the conductivity of the PLGA and PDA@PLGA membranes cannot be measured. After the addition of gold nanoparticles, the conductivity of the membrane materials increased to 0.039 ± 0.006 S/cm, indicating that gold nanoparticles can improve the conductivity of membrane materials.

### Antimicrobial properties of the Os/Au-PDA@PLGA composite membrane

**Fig. 4:**
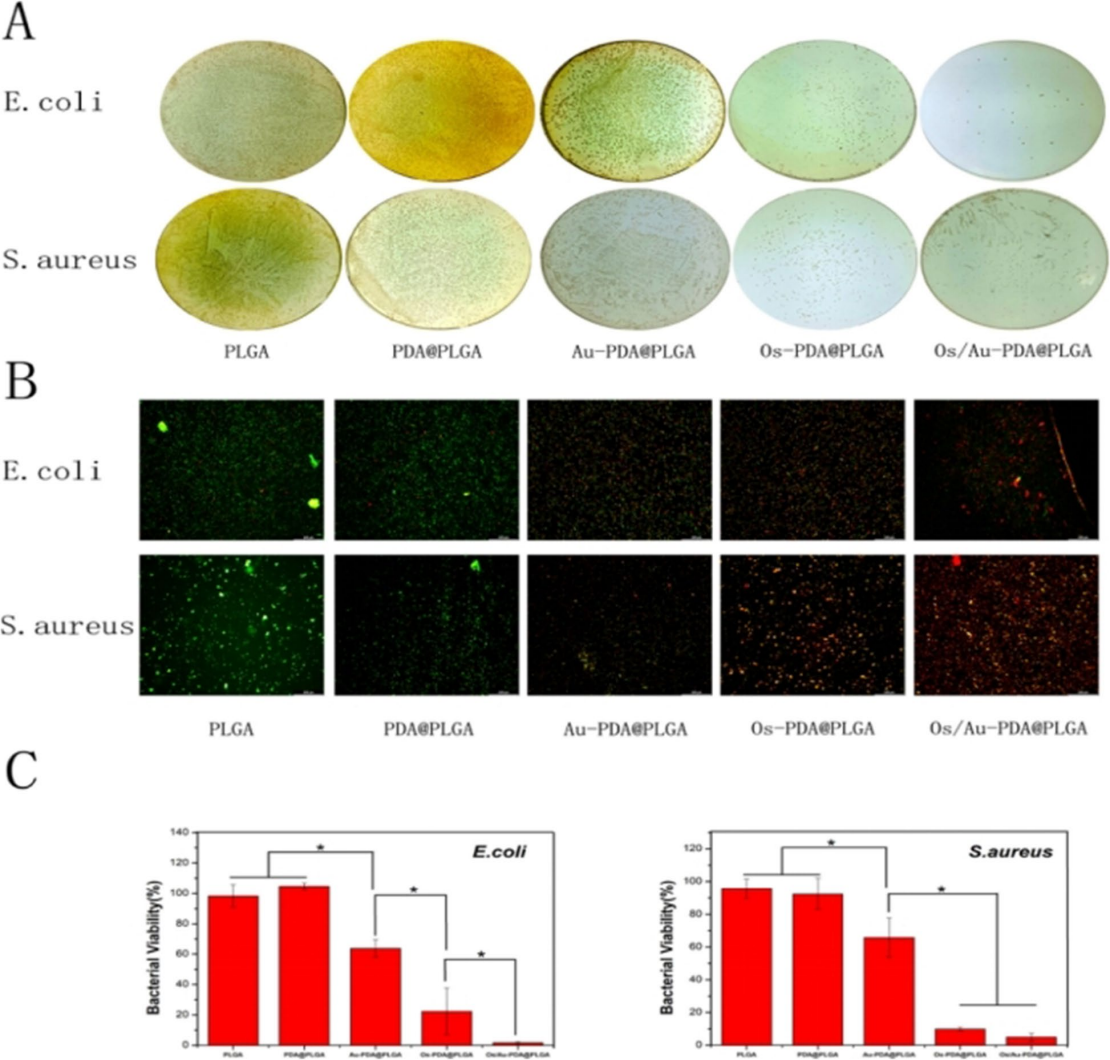
Colony growth (**A**) and bacterial viability (**C**) of *E*. *coli* and *S. aureus* cultured in different composite membranes. (**B**) Bacterial live and dead staining after co-culture with different composite membranes. Green and red represent live and dead bacteria, respectively. *p* < 0.05, *n* = 3, Bar lengths are 100 μm

**Fig. 5:**
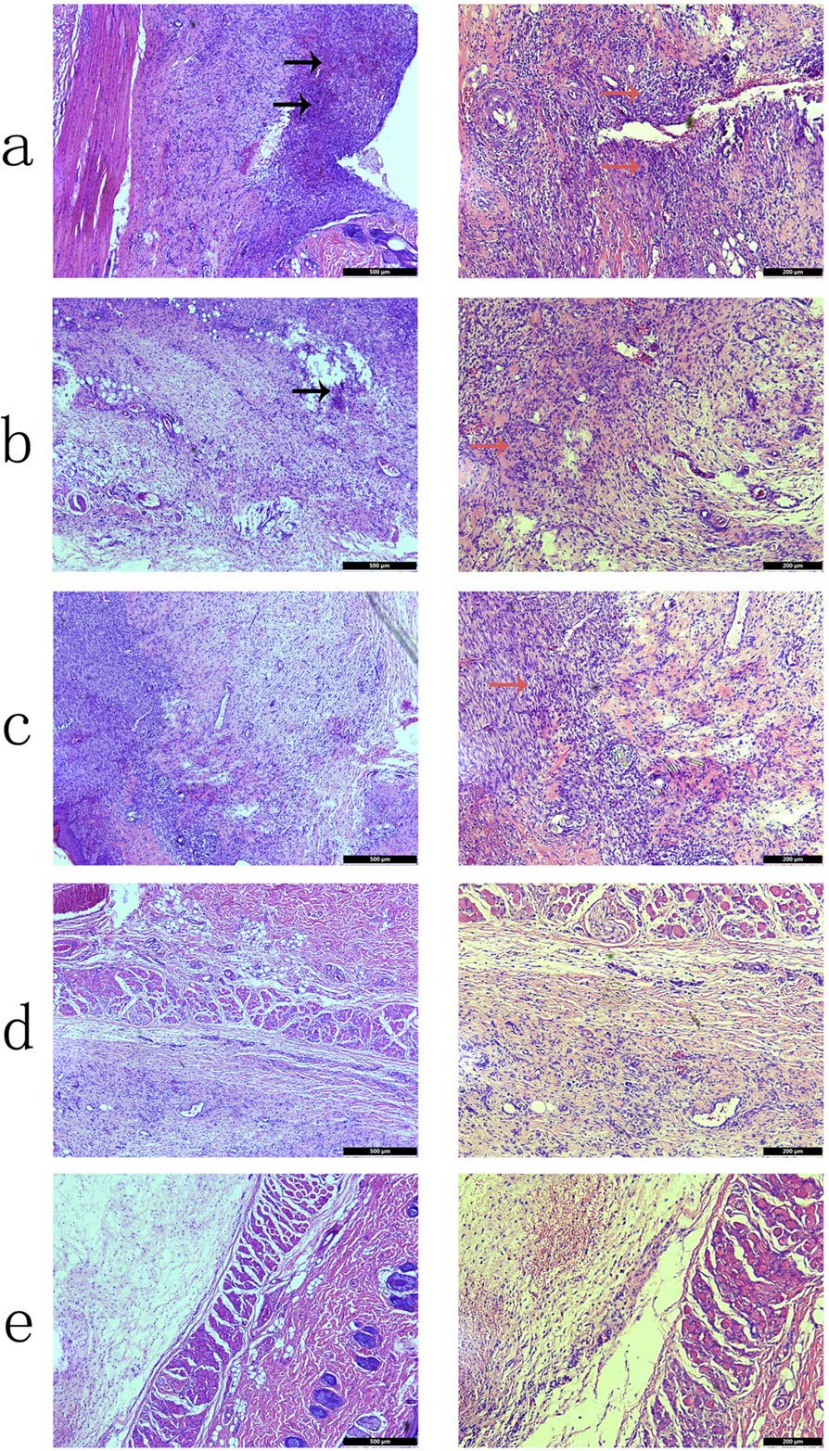
H&E staining of the skin tissue embedded with different composite membranes after the wound site was infected with bacteria: (**a**) PLGA, (**b**) PDA@PLGA, (**c**) Au-PDA@PLGA, (**d**) Os-PDA@PLGA and (**e**) Os/Au-PDA@PLGA. Bar lengths are 500 μm (left) and 200 μm (right). Black arrow: necrotic tissue, Orange arrow: Neutrophils, lymphocytes and other immune cells

Bacterial infection is one of the important factors affecting wound repair. However, most polymer materials have low antibacterial capacity and therefore do not inhibit bacterial infection during wound closure. Therefore, in this study, we loaded gold nanoparticles and the antibacterial polypeptide Os on the surface of PLGA to improve the antibacterial properties of the material. As shown in Fig. 4A, a large number of colonies were observed on cell culture dishes in the PLGA and PDA@PLGA groups. In the Au-PDA@PLGA group, the number of colonies decreased slightly. The Os-PDA@PLGA and Os/Au-PDA@PLGA groups were better at reducing the number of bacteria than the Au-PDA@PLGA groups. Among all the groups, the Os/Au-PDA@PLGA group had the best antibacterial effect, and almost no bacterial colonies were observed on the cell culture dish. Bacterial viability results (Fig. 4C) showed that there was no significant difference between the PLGA and PDA@PLGA groups, while the survival rate of bacteria in the Au-PDA@PLGA group was significantly decreased. The survival rate of bacteria in OS-PDA@PLGA was less than 30%, and the survival rate in Os/Au-PDA@PLGA further decreased to less than 10%. Subsequently, the bacterial solution was cocultured with different membrane materials, and the antibacterial properties of the membrane materials were evaluated by live and dead bacteria staining methods. The staining results of live and dead bacteria are shown in Fig. 4B, where green is live bacteria and red is dead bacteria. The results showed that a large number of dead bacteria were found in the Au-PDA@PLGA and Os/Au-PDA@PLGA groups. Next, we evaluated the antibacterial activity of different membrane materials against bacterium-infected tissues. As shown in Fig. 5, H&E staining showed a large number of necrotic tissues in the PLGA, PDA@PLGA and Au-PDA@PLGA groups, with diffuse distribution of lymphocytes, neutrophils, and macrophages. After the addition of Os, the necrotic area of tissue was significantly reduced, and the phenomena of nuclear fragmentation and dissolution were also significantly reduced. Especially in the Os/Au-PDA@PLGA group, only a small number of inflammatory cells invaded the tissue, and the antibacterial effect of the membrane material was the most obvious. According to the above experimental results, the antibacterial polypeptide Os can significantly improve the antibacterial activity of PLGA materials. More importantly, the addition of gold nanoparticles can further enhance the resistance of bacteria to Os.

### ROS scavenging effect of the Os/Au-PDA@PLGA Composite membrane

**Fig. 6:**
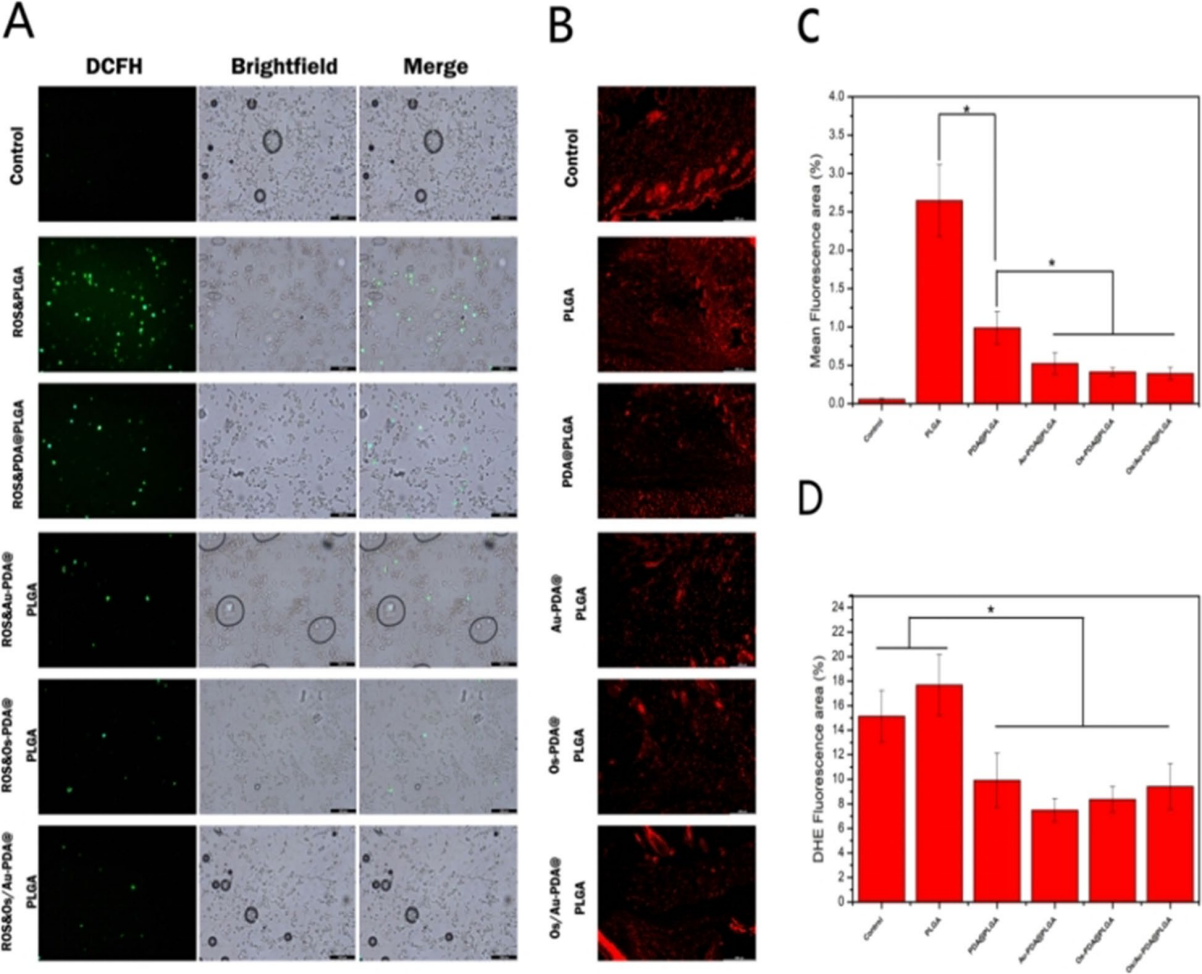
(**A**) Intracellular ROS levels of NIH3T3 cells after oxidative stimulation. Scale bar = 200 μm. (**B**) ROS scavenging of different composite membranes in vivo. Scale bar = 500 μm. (**C**) Mean fluorescence area of ROS in different groups. (**D**) Mean fluorescence intensity of DHE in different composite membranes. *p* < 0.05, *n* = 3

Excessive inflammation and ROS can prolong the inflammatory period during wound healing, resulting in delayed or non-healing wound [32]. Therefore, wound repair materials should have certain ROS scavenging abilities. In this study, to evaluate the ROS scavenging effect of different membrane materials, various membrane materials were incubated with NIH3T3 cells stimulated by H_2_O_2_. As shown in Fig. 6A, after H_2_O_2_ stimulation, excessive ROS could be observed in the PLGA group compared with the normal NIH3T3 cells. Compared with the PLGA group, the ROS levels in the PDA@PLGA groups were significantly reduced, which was due to the ROS elimination property of the phenol hydroxyl group in PDA [33]. After the addition of Os and gold nanoparticles, ROS-positive cells were further reduced. Some studies have found that the antioxidant activity of the antibacterial polypeptide Os was due to the presence of free cysteine residues where the sulfhydryl group interacts with the radical species by hydrogen donation from the SH group [34]. Furthermore, gold nanoparticles also have a strong antioxidant effect, which can clear ROS and inhibit inflammation after injury [35]. Among all the groups,the mean fluorescence area reuslts (Fig. 6C) show the Os/Au-PDA@PLGA membrane had the best ROS scavenging effect in vitro. The results of DHE staining in vivo showed a similar trend. As shown in Fig. 6B, DHE staining showed that the control group and the PLGA group had strong signals, indicating that the wound had a high ROS content. In contrast, a decrease in the staining intensity in the PDA@PLGA-treated group indicates a decrease in ROS content at the wound. The positive expression of DHE in Au-PDA@PLGA, Os-PDA@PLGA and Os/Au-PDA@PLGA group was the lowest (Fig. 6D). The above results indicate that the Os/Au-PDA@PLGA composite membrane has a good scavenging effect on ROS, and this property can reduce the inflammatory response when applied in skin wound repair.

### Cell proliferation, adhesion and migration assay

**Fig. 7:**
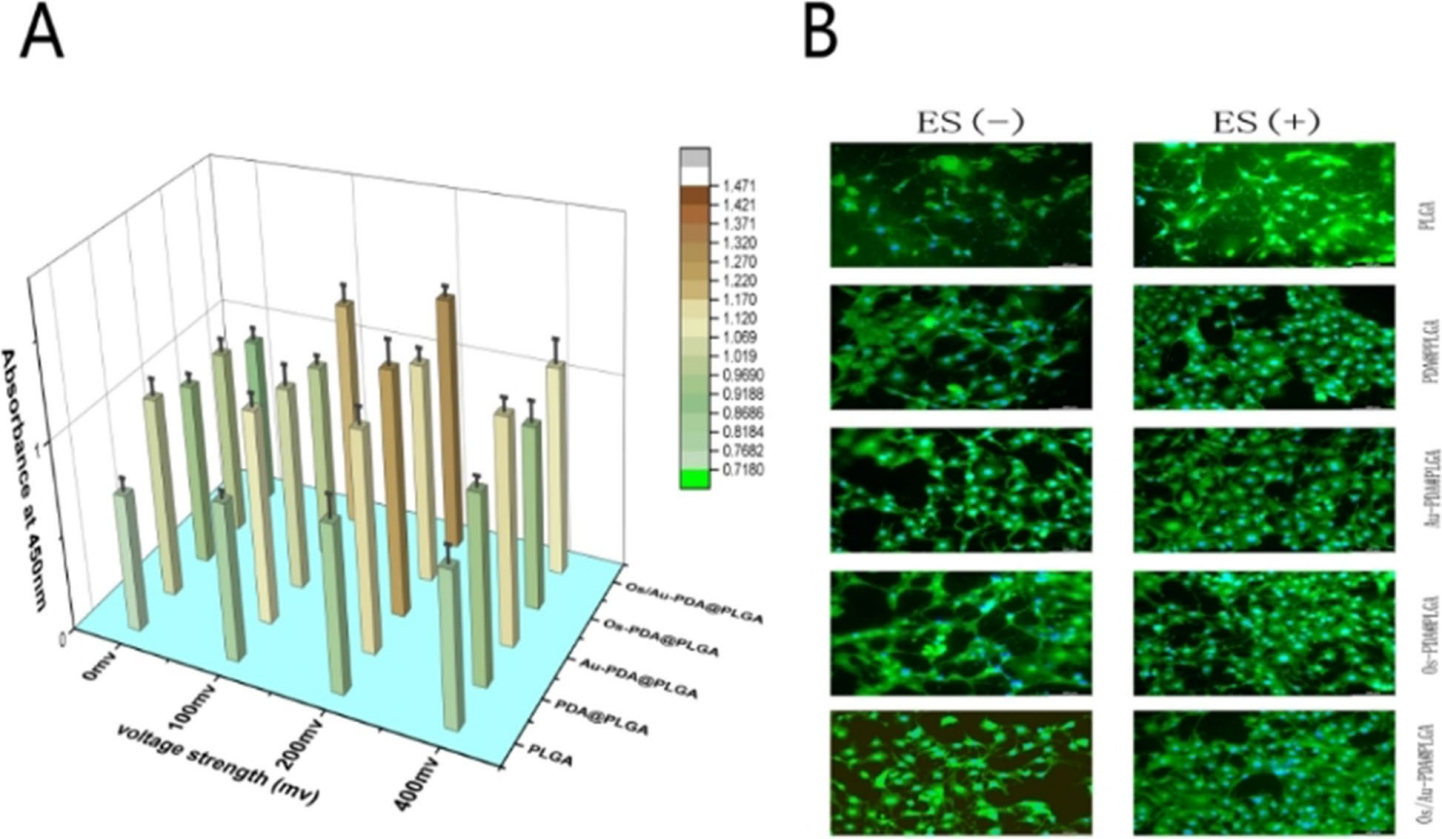
(**A**) Cell proliferation after 4 days of culture. (**B**) Fluorescent images of NIH3T3 cells on day 4. Scale bar = 100 μm, *p* < 0.05, *n* = 3

In this study, we evaluated the effect of a conductive Os/Au-PDA@PLGA membrane and electrical stimulation on cell activity with a homemade device. To determine appropriate electrical stimulation parameters, a CCK-8 assay was used to detect the proliferation of NIH3T3 cells under different treatment conditions. As shown in Fig. 7A, on day 4, the OD values in the ES group were higher than those in the control group, and the highest OD values were detected in the 200 Hz group. To test the effect of the combined effect of ES and different membrane materials on the proliferation of cells. NIH3T3 cells were implanted on different membranes with or without ES. The number of cells in the PDA@PLGA groups was higher than that in the PLGA group (p < 0.05), which indicated that the PDA coating could effectively promote cell proliferation. However, after loading gold nanoparticles on the membrane surface, the cell proliferation rate decreased slightly (*P* > 0.05). There was no significant change in cell proliferation after the addition of Os, which indicated that Os had no obvious cytotoxicity. After electrical stimulation, the cell proliferation rate of each group was improved to varying degrees. However, cell proliferation was more pronounced in the group containing gold nanoparticles. After 3 days, the cell OD values of Au-PDA@PLGA + ES and Os/Au-PDA@PLGA + ES were significantly higher than those in other groups. To further observe the effect of different membrane materials and electrical stimulation on cell adhesion, we observed cell adhesion and diffusion on different membrane material surfaces. As shown in Fig. 7B, although the cells on the surface of the pure PLGA material grew well, the number of cells was lower, and most of the cells did not spread out. Compared with the PLGA group, the number of adherent cells on the PDA@PLGA membrane surface was greater, and the adherent area was larger. Compared with the PDA@PLGA group, there was no significant difference in cell morphology between the Au-PDA@PLGA, Os-PDA@PLGA and OS/Au-PDA@PLGA groups. Furthermore, electrical stimulation could modulate the morphology of cells cultured on different membrane materials. The adhesion area and number of cells cultured on PLGA, PDA@PLGA and Os-PDA@PLGA membranes increased slightly under electrical stimulation. However, cells on the electroactive Au-PDA@PLGA and Os/Au-PDA@PLGA membranes differed significantly in response to electrical stimulation. In the Os/Au-PDA@PLGA + ES and Au-PDA@PLGA group + ES group, the number and area of adherent cells was larger, the diffusion state was better, the cells were almost completely stacked on each other, and the interaction between cells was more prominent, indicating that the cells had strong adhesion behavior. The above results further supported the role of the combined application of Os/Au-PDA@PLGA composite membranes and electrical stimulation in promoting the attachment and proliferation of cells.

**Fig. 8:**
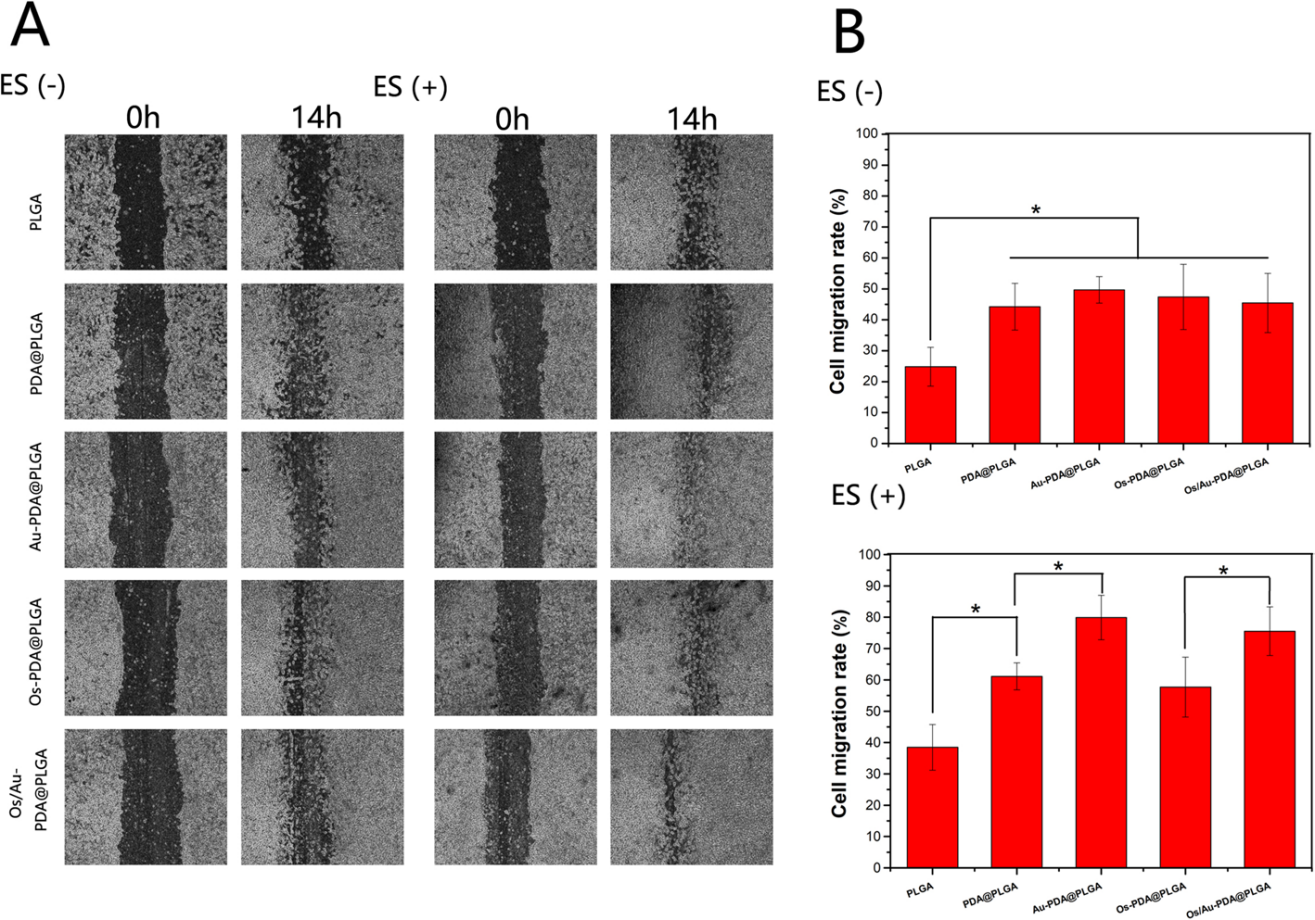
(**A**) Optical microscope images of wounded areas after 14 h of incubation. (**B**) Wound healing rate in each group without/with electrical stimulation. *p* < 0.05, *n* = 3

Fibroblast migration plays an important role in wound re-epithelialization and closure. The ability of different membrane materials and ES to promote cell migration was studied by a cell scratch assay. To ensure that cell migration was not caused by cell proliferation, we serum-starved the cells 24 h before the scratch test to reduce cell proliferation. After 14 h of treatment, the closure result of the scratched area showed significant differences between the PDA@PLGA group and the PLGA group (Fig. 8A). The PDA@PLGA membrane was able to induce NIH3T3 cells to migrate at a significantly faster rate in scratches, whereas cells in the PLGA group showed slower migration. In the Au-PDA@PLGA, Os-PDA@PLGA, and Os/Au-PDA@PLGA groups, NIH3T3 cells showed similar migration ability as those in the PDA@PLGA group. The migration ability of cells in each group was further enhanced after electrical stimulation. Among all groups, the Os/Au-PDA@PLGA + ES and Au-PDA@PLGA + ES groups had the strongest promoting effect on cell migration. More specifically, the relative scratch widths of the Os/Au-PDA@PLGA + ES and Au-PDA@PLGA + ES groups were almost completely closed at 14 h. Quantitative analysis of the cell scratch test showed that the scratch healing rates of the Os/Au-PDA@PLGA + ES and Au-PDA@PLGA + ES groups were 75.54 ± 7.75% and 79.9 ± 7.09%, respectively. The above results indicate that the Os/Au-PDA@PLGA composite membrane combined with electrical stimulation can significantly promote the migration ability of cells.

### In vivo wound healing assessment

**Fig. 9:**
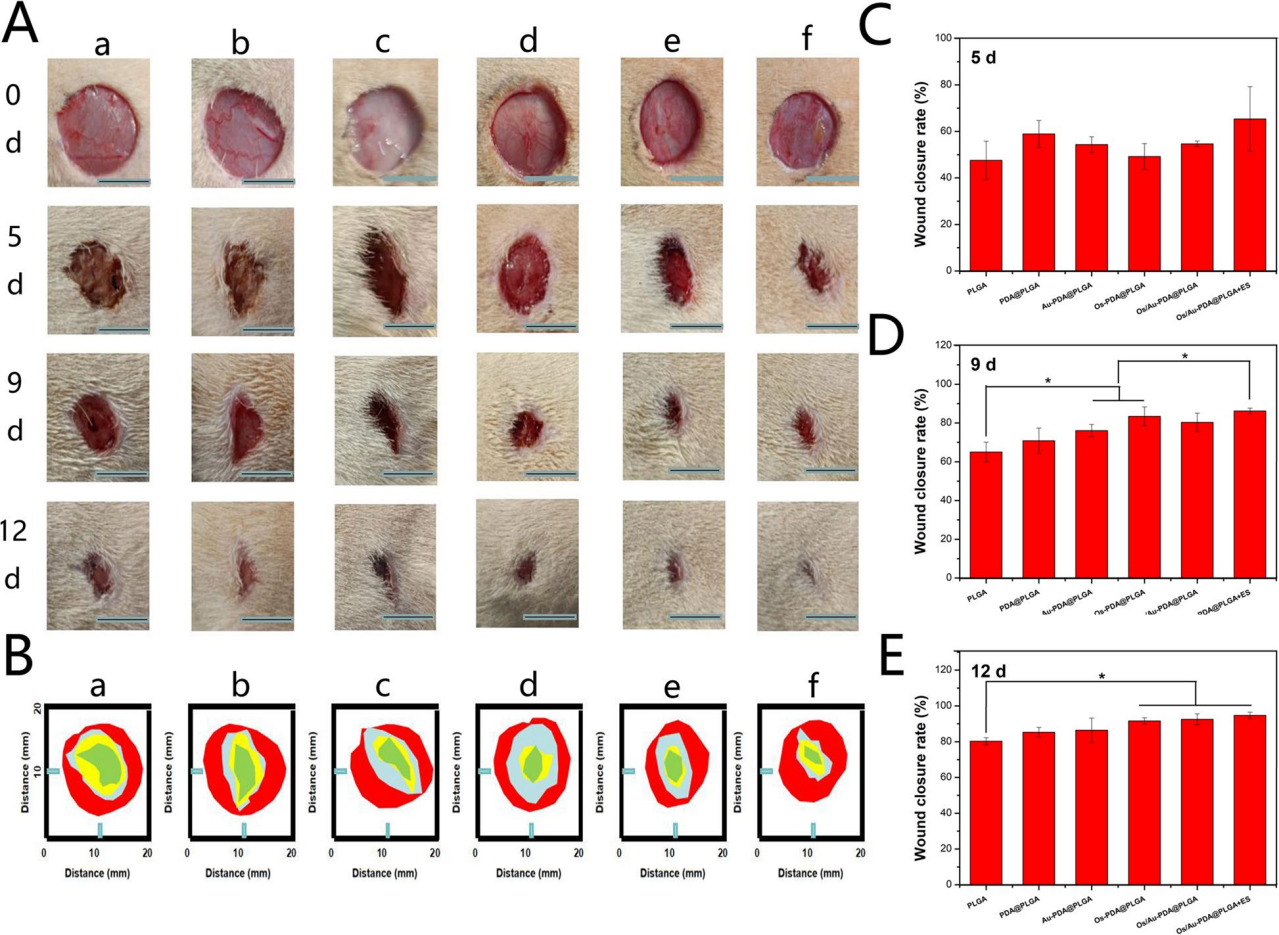
(**A**) The closure rate of the wound defects with different treatments at days 0, 5, 9, and 12. (**B**) Schematic diagram of wound closure at different time points. (a) PLGA, (b) PDA@PLGA, (c) Au-PDA@PLGA, (d) Os-PDA@PLGA, (e) Os/Au-PDA@PLGA and (f) Os/Au-PDA@PLGA + ES. (**C**-**E**) Quantitative statistical analysis of wound closure for different treatments at 5, 9 and 12 days. Scale bar = 1 cm, *p* < 0.05, *n* = 3

To verify the efficacy of different membrane materials and electrical stimulation in vivo, a rat skin defect model was used to evaluate the effects of membrane materials and electrical stimulation on skin wound healing. Representative pictures of the wounds in each group at scheduled time intervals are displayed in Fig. 9A and B. With the extension of time, the wound size of each group tended to decrease. Macroscopically, significantly accelerated wound closure was observed in the other groups as early as day 5 compared to the PLGA group. On day 9, the wound healing rate was the lowest in the PLGA group, with a large scab observed on the wound, followed by the PDA@PLGA and Au-PDA@PLGA groups. Compared with the PDA@PLGA and Au-PDA@PLGA groups, the skin wound areas in the Os-PDA@PLGA and Os/Au-PDA@PLGA groups were smaller, and the healing effect was better. We speculated that the antimicrobial peptides could effectively improve the antibacterial properties of the composite membrane and inhibit the infection caused by pathogens, thus creating a better healing environment for wound healing. Among all groups, the Os/Au-PDA@PLGA + ES group had the fastest reduction in wounds and the best closure rate (Fig. 9C-E). The wounds of the Os/Au-PDA@PLGA + ES group were almost completely closed after 12 days of treatment. As shown in Fig. 9E, after 12 days of treatment, the wound closure rate in the Os/Au-PDA@PLGA + ES groups was significantly higher than that in the other groups, reaching 94.76%. The above results showed that ES treatment combined with the Os/Au-PDA@PLGA composite membrane is more effective than the Os/Au-PDA@PLGA composite membrane in promoting the healing of full-thickness skin defects in rats.

### Histologic analysis

**Fig. 10:**
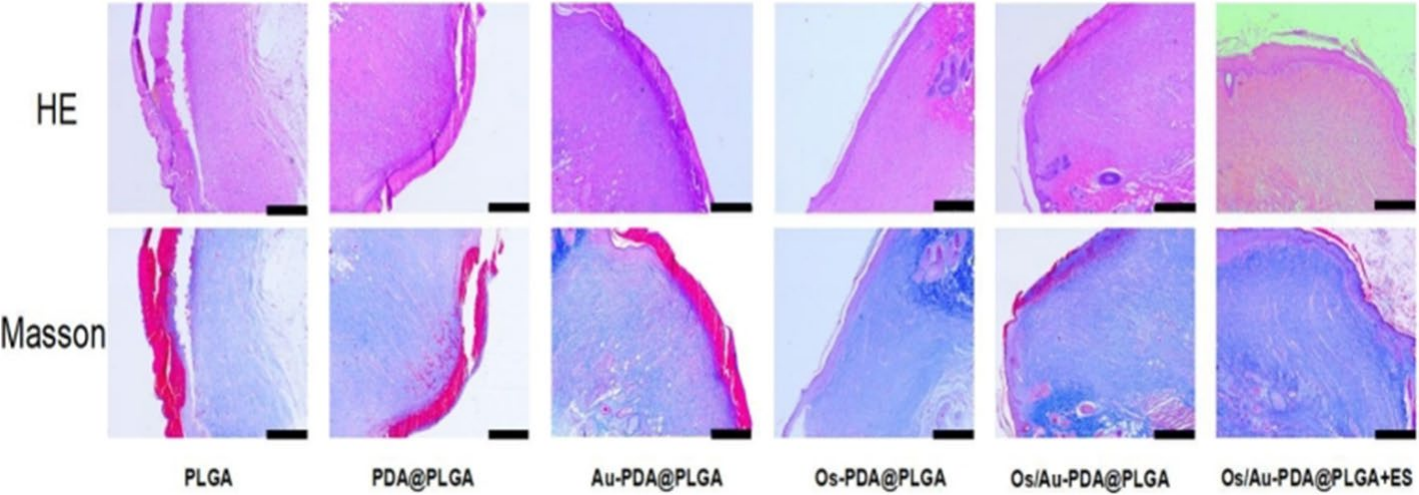
Histological appearance of wounds harvested on day 12 in each group. Scale bar = 500 μm

To further determine the tissue repair capability of the scaffold, we performed histological analysis of the skin healing tissue. As shown in Fig. 10, HE staining and Masson staining were used to evaluate the regeneration of skin tissue after trauma. At the beginning of skin healing, the epidermis gradually thickens to a greater than normal thickness as cells proliferate. As healing continued, the skin thickness gradually returned to normal. Thus, the degree of reepithelialization is considered to be an appropriate evaluation criterion for wound repair. In all groups, the skin tissue sections lacked skin appendages such as hair follicles. The HE staining results showed that the skin thickness of the PLGA group was the largest, while that of the Os/Au-PDA@PLGA + ES group was the smallest, which proved that the skin repair effect of Os/Au-PDA@PLGA + ES was better. Wound healing was improved in the PDA@PLGA group compared to PLGA group; however, the regenerated dermis was not as dense as normal tissue. Tissues in the Os-PDA@PLGA and Os/Au-PDA@PLGA groups showed efficient skin layer regeneration. The overall tissue structure of the Os-PDA@PLGA and Os/Au-PDA@PLGA groups was more compact, and the tissue inflammatory cell infiltration was significantly reduced, indicating that the continuous release of Os had a significant bactericidal effect and promoted the healing of skin wounds. Compared to the other groups, the Os/Au-PDA@PLGA + ES group had the densest connective tissue, similar to normal rat skin tissue. Next, Masson staining was performed on new skin tissue to detect collagen deposition in the wound healing area. According to the Masson staining results, collagen in the wound skin of the PLGA group was sparse and disordered. The collagen staining intensity of the PDA@PLGA, Au-PDA@PLGA and Os-PDA@PLGA groups was higher than that of the PLGA groups. Among all groups, the Os/Au-PDA@PLGA and Os/Au-PDA@PLGA + ES groups had the highest density, thickness and optimal arrangement of collagen deposition.

**Fig. 11:**
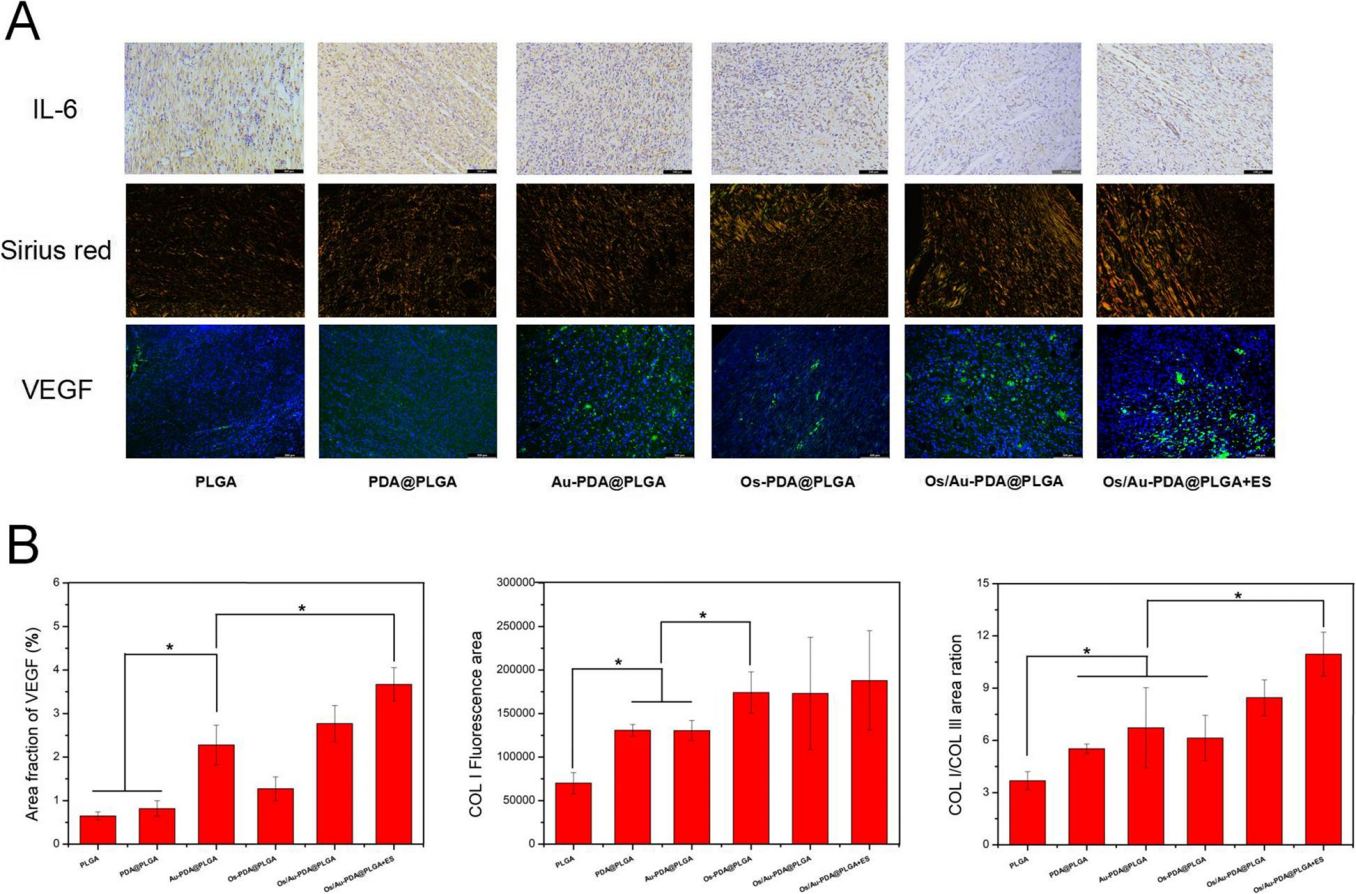
(**A**) IL-6, Sirius red and VEGF staining in the wound-healing region at 12 days after different treatments. (**B**) Quantitative statistical analysis of VEGF, COL I relative area percentage and COL I/COL III area ratio in the wound-healing region. Scale bar = 100 μm (IL-6 and VEGF), Magnification, 200 × (Sirius red), *p* < 0.05, *n* = 3

Collagen is the main component of skin tissue, and there are two main kinds of collagen: type I collagen (COL-I) and type III collagen (COL-III). At the initial stage of wound healing, the content of COL-III in tissue was high. As the skin tissue continues to heal, COL-III is gradually replaced by COL-I to increase the tensile strength of the skin [36]. In this study, the Sirius red staining method was used to analyse the collagen types on the wound (COL-I: red/orange and COL-III: blue). As shown in Fig. 11A, a higher proportion of COL-III was found in the PLGA group, indicating poor wound healing. COL-I expression was increased in the PDA@PLGA and Au-PDA@PLGA groups compared with the PLGA group. In the Os/Au-PDA@PLGA + ES group, the COL-I content in the skin wound samples was further increased, indicating that Os/Au-PDA@PLGA + ES greatly improved the wound healing speed.

Angiogenesis is a key factor in skin wound repair. New blood vessels can not only attract monocytes to the wound site but also provide oxygen and growth factors needed for tissue healing, thus accelerating wound healing [37]. VEGF is considered to be an endothelial marker of new capillary formation [38]. In this study, wound angiogenesis was evaluated by VEGF immunofluorescence staining. As shown in Fig. 11A, the VEGF average fluorescence density in the PLGA group was the lowest among all groups. The VEGF average fluorescence density was not significantly different between the PDA@PLGA and Os-PDA@PLGA groups (Fig. 11B). The expression of VEGF in the Au-PDA@PLGA and Os/Au-PDA@PLGA groups was significantly higher than that in the other groups. Among all groups, the Os/Au-PDA@PLGA + ES group had the largest stained area of VEGF, indicating a significant increase in angiogenesis, further demonstrating the synergic effect of electrical stimulation and the Os/Au-PDA@PLGA composite membrane in the formation of new blood vessels. The inflammatory response caused by skin injury is also an important factor affecting wound healing. IL-6 is a proinflammatory cytokine that can be used as a marker of inflammation. In this study, IL-6 immunohistochemical staining was used to evaluate the inflammation level at the wound site. As shown in Fig. 11, a large amount of IL-6-positive tissue was found in the wounds of the PLGA and PDA@PLGA groups. Compared with the PLGA and PDA@PLGA groups, IL-6-positive tissue was significantly reduced in the Au-PDA@PLGA and Os-PDA@PLGA groups. In the Os/Au-PDA@PLGA and Os/Au-PDA@PLGA + ES groups, IL-6-positive tissue was further reduced, indicating that the loading of gold nanoparticles and Os could effectively improve the anti-inflammatory activity of the membrane materials.

**Fig. 12:**
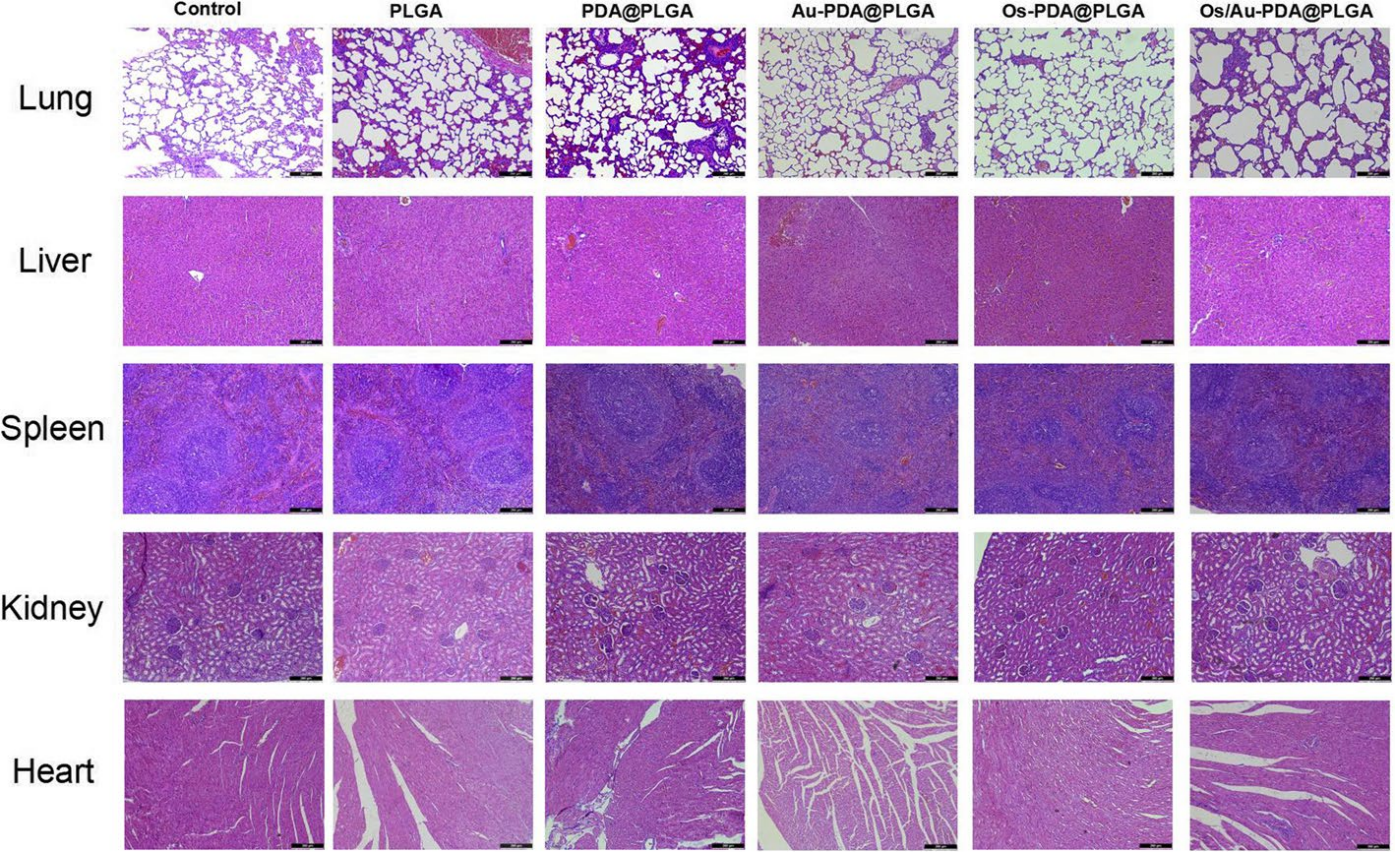
H&E staining sections of important organs (lung, liver, spleen, kidney and heart) in experimental animals. Scale bar = 200 μm

According to the skin wound healing rate and histological analysis, we found that the Os/Au-PDA@PLGA composite membrane has good skin tissue repair effects. However, gold nanoparticles and other nanomaterials can enter important organs of the body through the blood circulation, and the long-term toxicity of antibacterial peptides in vivo is less studied. Therefore, the biosafety of Os/Au-PDA@PLGA composite membranes also needs to be evaluated before their application. We implanted different composite membrane materials into the muscle of rats for one month and then removed the heart, liver, spleen, lung and kidney of experimental rats for HE staining to evaluate the biotoxicity of the materials. As shown in Fig. 12, no significant pathological changes were found in the important internal organs of rats in each experimental group compared with the normal rats, and the internal organs were in normal shape. The following results of HE on the organs of experimental animals showed that the prepared Os/Au-PDA@PLGA composite membranes did not cause obvious chronic toxicity in vivo.

Previous studies have focused on improving tissue angiogenesis or preventing bacterial infections when treating skin wounds [1, 39]. However, these effects are limited due to the complex pathological environment at the skin wound site. The most fundamental causes of nonhealing skin wounds are oxidative stress, inflammation and infection caused by the wound microenvironment. Therefore, eliminating oxidative stress, inflammation and infection is an effective strategy to accelerate skin wound healing. The antibacterial polypeptide Os has good antioxidant, anti-inflammatory and antibacterial effects and is an ideal substitute for antibiotics [40]. Gold nanoparticles are also commonly used in skin tissue repair and have excellent electrical activity and can improve the activity of antibacterial drugs [41]. Moreover, gold nanoparticles had excellent anti-inflammatory and antioxidant effects, which could effectively remove ROS and inhibit oxidative stress damage. In this study, Os and gold nanoparticles were selected as bioactive factors and loaded on the surface of a PLGA membrane by PDA coating to prepare a wound repair material with excellent antibacterial, anti-inflammatory, biocompatibility and tissue repair ability and realize efficient healing of skin wounds.

In this study, we found that the Os/Au-PDA@PLGA composite membrane showed an excellent ability to accelerate skin wound healing, mainly for the following reasons. First, PDA coatings can effectively improve the hydrophilicity and cytocompatibility of materials, creating a better extracellular microenvironment for cell growth and migration. The adhesion ability of PDA also promotes the deposition of nutrients around damaged tissues, further improving the healing rate of the skin. Furthermore, Os, as an antibacterial peptide, has been shown to have good antibacterial activity and does not cause any adverse effects on eukaryotic cells. The loading of this antibacterial polypeptide can effectively improve the antibacterial activity of wound repair materials, inhibit the growth of pathogens, and eliminate the adverse factors affecting the healing of skin tissue. More importantly, Os can further enhance its antibacterial effect when used in combination with gold nanoparticles. Some have found that considerably enhanced antibacterial performance could be obtained even when minimal antibacterial drugs were applied with gold nanoparticles compared to the large antibacterial drug quantities used without gold nanoparticles [42]. The oxidative stress environment at the wound site can produce ROS, free radicals and other active substances to resist the invasion of foreign bodies and external stimuli [43, 44]. However, the excess presence of ROS can cause wound cells to age and induce an inflammatory response that prevents wound regeneration. In this study, we chose gold nanoparticles and Os, which also have excellent antioxidant effects. Our results show that the Os/Au-PDA@PLGA composite membrane has the ability to remove ROS during tissue regeneration due to the incorporation of gold nanoparticles and Os. Implantation of the antioxidant Os/Au-PDA@PLGA composite membrane can reduce the number of ROS in the wound. Elimination of ROS leads to a decrease in cell aging and reduces the inflammatory response, consequently promoting wound healing.

Endogenous DC electric fields generated by epithelial potential differences play an important role in regulating cell behavior during skin wound healing. Therefore, the application of exogenous electrical stimulation to the skin wound site is an effective strategy to accelerate wound healing. To maximize the effect of electrical stimulation, the collaborative use of electroactive materials and electrical stimulation has been a common method in recent years. In this study, the addition of gold nanoparticles endows the composite membrane with good electrical conductivity, which in turn leads to the electroactive composite membrane acting as a bridge for the transmission of electrical signals. Previous studies have shown that electroactive materials promote the expression of tissue repair-related genes [6, 45]. Cell adhesion and migration are regulated by functional proteins, and appropriate electrical signals can specifically regulate protein behavior. Our results showed that the cell proliferation, adhesion and migration in the Os/Au-PDA@PLGA + ES group were significantly better than those in the Os/Au-PDA@PLGA group, which had certain positive effects on cell behavior. In vivo full-thickness skin defect models showed that the combination of Os/Au-PDA@PLGA composite membrane and electrical stimulation could maximize wound healing. In summary, the Os/Au-PDA@PLGA composite membrane has a good skin wound repair effect. These effects are directly attributable to the involvement of various active factors, including Os with broad-spectrum antibacterial and anti-inflammatory abilities, PDA coatings promoting cell adhesion and migration, and gold nanoparticles with good electrical activity and anti-inflammatory properties. Furthermore, electrical stimulation further enhanced the repair effect of the Os/Au-PDA@PLGA composite membrane and accelerated the healing of the skin tissue.

## Conclusions

In this study, we developed a multifunctional material for skin wound repair by using the reduction and adhesive properties of PDA to load gold nanoparticles and the antibacterial polypeptide Os onto the surface of a PLGA membrane. The addition of PDA, Os and gold nanoparticles endows the membrane material with multiple functions. The composite membrane has good hydrophilicity, biocompatibility and conductivity and can be used as an electroactive substrate to transmit electrical signals and regulate cell function. Furthermore, Os and gold nanoparticles in the composite membrane also have antibacterial and antioxidant effects, which are conducive to the membrane material to inhibit bacterial growth and remove excessive ROS. More importantly, the combination of this multifunctional composite membrane with ES can simultaneously exert a variety of biological activities, including cell migration, angiogenesis and collagen deposition, promoting good healing of skin wounds. In conclusion, this new strategy of multifunctional composite membranes combined with electrical stimulation shows promise for use in clinical applications for the treatment of skin wounds.

### Supplementary Information

**Additional file 1: Figure S1.** Swelling rate of different membrane materials in PBS.

## Data Availability

The raw data used and/or analysed during the current study are available from the corresponding author upon reasonable request.

## Abbreviations

ES: Electrical stimulation
PLGA: Poly(lactic-co-glycolic acid)
PDA: Polydopamine
Au: Gold nanoparticles
ROS: Reactive oxygen species
rGO: Reduced graphene oxide
*S.aureus*: *Staphylococcus aureus*
*E.coli*: *Escherichia coli*
XPS: X-ray photoelectron spectroscopy
SEM: Scanning electron microscope
