# Supplementary material for: Mussel-inspired electroactive, antibacterial and antioxidative composite membranes with incorporation of gold nanoparticles and antibacterial peptides for enhancing skin wound healing
Source: J Biol Eng. 2024 Jan 11;18:3. doi: 10.1186/s13036-023-00402-3 (PMC10785445; doi:10.1186/s13036-023-00402-3)

**Support information for**

**Mussel-Inspired Electroactive,** [**Antibacterial**](javascript:;) **and** **Antioxidative Composite Membranes with Incorporation of Gold Nanoparticles and Antibacterial Peptides for Enhancing Skin Wound Healing**

Yongkang Dong^1,2^, Zheng Wang^2^, Jiapeng Wang^3^, Xuedi Sun^1^, Xiaoyu Yang^1*^, Guomin Liu ^1*^

^1^ Department of Orthopaedic Surgery, The Second Hospital of Jilin University, Changchun 130021, China

^2^ Department of Vascular Surgery, The Second Hospital of Jilin University, Changchun 130021, China

^3^ Department of Orthopaedic Surgery, Jilin Province FAW General Hospital, Changchun 130000, China

*** Correspondence:**

Guomin Liu

Department of Orthopaedic Surgery, The Second Hospital of Jilin University, Changchun 130021, China

1. mail: [liuyedao123@163.com](mailto:liuyedao123@163.com)

Xiaoyu Yang

Department of Orthopaedic Surgery, The Second Hospital of Jilin University, Changchun 130021, China

E-mail: yangxiaoy@jlu.edu.cn

**Supplementary Figures**

**Fig. S1 Swelling rate of different membrane materials in PBS.**


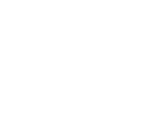

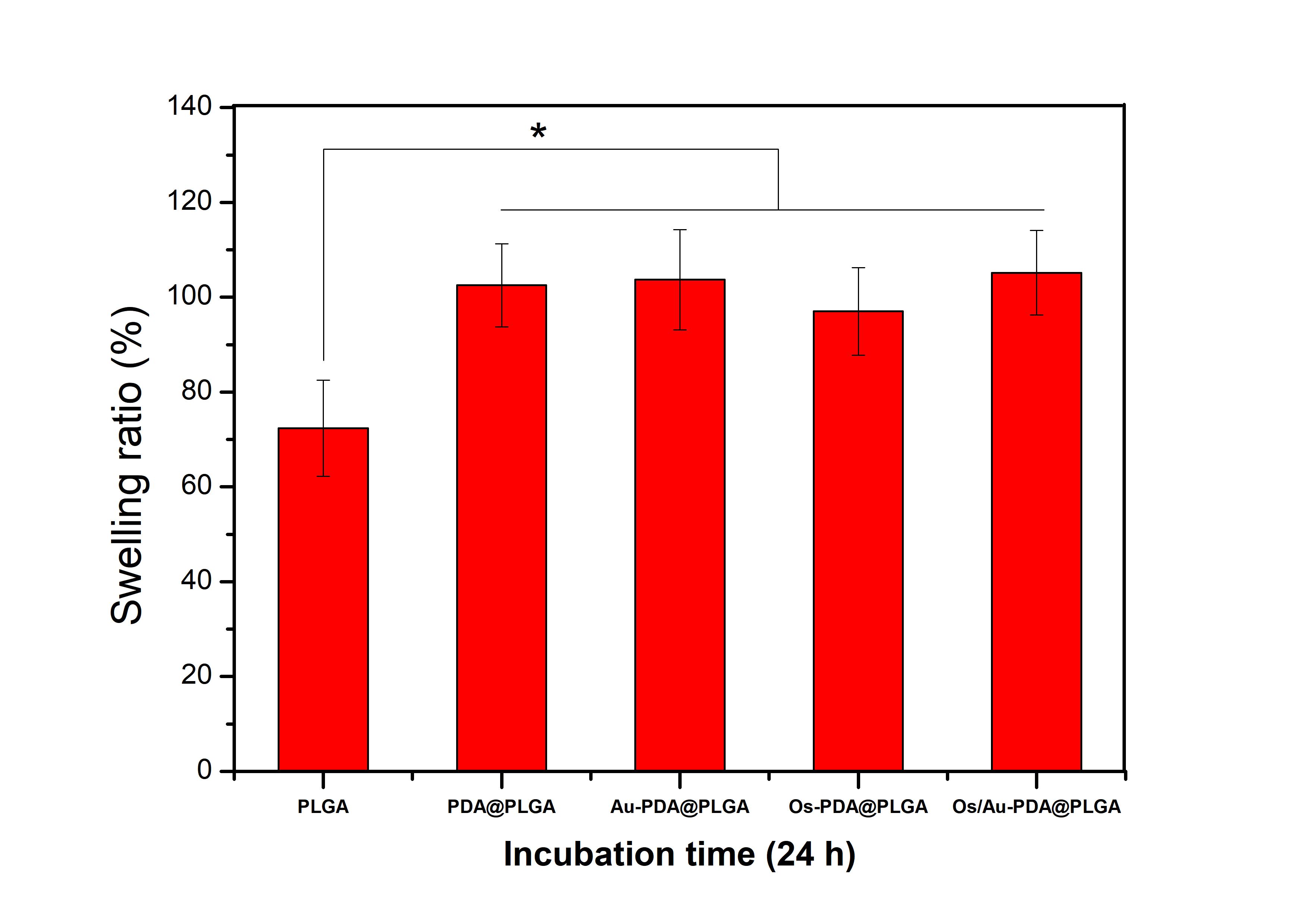

Supplement: Supplementary file 1 — Additional file 1: Figure S1. Swelling rate of different membrane materials in PBS. [file 13036_2023_402_MOESM1_ESM.doc]
